# CPP-GMR Performance of Electrochemically Synthesized Co/Cu Multilayered Nanowire Arrays with Extremely Large Aspect Ratio

**DOI:** 10.3390/nano10010005

**Published:** 2019-12-18

**Authors:** Himeyo Kamimura, Masamitsu Hayashida, Takeshi Ohgai

**Affiliations:** 1 Graduate School of Engineering, Nagasaki University, Bunkyo-machi 1-14, Nagasaki 852-8521, Japan; bb52118618@ms.nagasaki-u.ac.jp; 2 Faculty of Engineering, Nagasaki University, Bunkyo-machi 1-14, Nagasaki 852-8521, Japan; hayashida@nagasaki-u.ac.jp

**Keywords:** electrodeposition, multilayer, nanowire, cobalt, copper, anodization, nanochannel, magnetization, magnetoresistance, CPP-GMR

## Abstract

Anodized aluminum oxide (AAO) films, which have numerous nanochannels ca. 75 nm in diameter, *D* and ca. 70 µm in length, *L* (ca. 933 in aspect ratio, *L*/*D*), were used as a template material for growing Co/Cu multilayered nanowire arrays. The multilayered nanowires with alternating Cu layer and Co layers were synthesized by using an electrochemical pulsed-potential deposition technique. The thickness of the Cu layer was adjusted from ca. 2 to 4 nm while that of the Co layer was regulated from ca. 13 to 51 nm by controlling the pulsed potential parameters. To get a Co/Cu multilayered nanowire in an electrochemical in-situ contact with a sputter-deposited Au thin layer, the pulsed potential deposition was continued up to ca. 5000 cycles until the nanowire reached out toward the surface of AAO template. Current-perpendicular-to-plane giant magnetoresistance (CPP-GMR) effect reached up to ca. 23.5% at room temperature in Co/Cu multilayered nanowires with ca. 3500 Co/Cu bilayers (Cu: 1.4 nm and Co: 18.8 nm). When decreasing the thickness of Co layer, the CPP-GMR value increased due to the Valet–Fert model in the long spin diffusion limit.

## 1. Introduction

Due to a uniaxial shape anisotropy and a large surface area, metallic nanowires with an extremely large aspect ratio have a variety of potential applications to novel functional devices [1,2,3,4]. Particularly, ferromagnetic metal nanowires are expected to be able to apply for a magnetic field sensor with anisotropic magnetoresistance response [5,6] or giant magnetoresistance response [7,8]. Metallic nanowires can be synthesized by a metal-catalyzed vapor-phase method [9] or a wet chemical etching method [10]. On the other hand, some researchers have reported that the nanowires can be also fabricated by the electrochemical growth of metallic crystals from an aqueous solution into nanochannel template materials [11,12,13]. The electrodeposition technique into numerous nanochannels has a large merit rather than the others with respect to the productivity of numerous nanowires with straight shape. Polymer-based nanochannel films [14,15] and aluminum-oxide-based nanochannel films [16,17,18] are well known to be able to use as a template material for electrochemical growth of nanowires. Several researchers have reported that the current-perpendicular-to-plane giant magnetoresistance (CPP-GMR) effect of metallic multilayered nanowires which were electrodeposited in anodized aluminum oxide (AAO) templates reached up to ca. 20% at room temperature [19,20,21]. Cox et al. reported that CPP-GMR effect reached up to 14% in FeCoNi/Cu multilayered nanowire networks with ca. 200 nm in diameter, *D* and ca. 15 µm in length, *L* (ca. 75 in aspect ratio, *L*/*D*) which were separated from commercially available AAO templates after the electrodeposition. In the paper, they have found that the sheet resistance, *R*_s_ of the multilayered nanowire networks was ca. 40 Ω/sq. [19]. Here, *R*_s_ = *ρ*/*t*_s_. *ρ* and *t*_s_ are defined to the resistivity and the film thickness. Davis et al. also reported that CPP-GMR effect reached up to 20% in CoNiFe/Cu multilayered nanowires, which were electrodeposited in commercially available AAO templates with ca. 200 nm in diameter, *D* and ca. 60 µm in length, *L* (ca. 300 in aspect ratio, *L*/*D*) [20]. Tang et al. reported that CPP-GMR effect reached up to 23% in CoNi/Cu multilayered nanowires, which were electrodeposited in commercially available AAO templates with ca. 250 nm in pore diameter, *D* and ca. 60 µm in pore length, *L* (ca. 240 in aspect ratio, *L*/*D*). In the report, it was revealed that the resistance of the multilayered nanowires was less than 100 Ω [21]. It is well known that GMR value, *G_MR_* is defined by the following Equation (1) [21].

(1) $$G_{MR}=\frac{R^{AP}-R^{P}}{R^{P}}$$

Here, *R^AP^* is the CPP resistance of a multilayer with the magnetization vectors of successive ferromagnetic layers aligned antiparallel, while *R^P^* is the corresponding resistance with them aligned parallel. Usually, an electrical contact process is employed by using the nanowires samples which are taken out from an electrolytic solution after the electrochemical growth of the nanowires. Hence, the surface of the metallic nanowires will be oxidized by an atmospheric circumstance. Furthermore, if the nanowires are made of an electrical contact with a lead layer by using a commercially available conductive paste, the following resistance measurement will be unstable due to the phase transformation of chemical substances in the paste. These metal oxidation and unstable conductive materials will cause a resistance which is not originated from the metallic nanowires. When the contact resistance between nanowires and a lead layer, *R_cont_* is introduced to the above GMR definition, Equation (1) can be transformed to the following Equation (2).

(2) $$G_{MR}^{*}=\frac{\left(R^{AP}+R_{cont}\right)-\left(R^{P}+R_{cont}\right)}{\left(R^{P}+R_{cont}\right)}=\frac{R^{AP}-R^{P}}{R^{P}+R_{cont}}$$

If the resistance of nanowires (*R^AP^* and *R^P^*) is larger than the contact resistance (*R_cont_*), the effect of *R_cont_* will be reduced and a precise resistance measurement to determine the GMR value will be realized. Wegrowe et al. reported that the electrochemical in-situ contact process was effective to realize the stable resistance measurement for electrodeposited metallic nanowires [22]. In the in-situ contact process, a nanochannel membrane film with a thin conducting layer was used as a template for electrodeposition of nanowires and the electrochemical in-situ contact was realized when the first nanowires reached the top conducting surface. However, they used commercially available ion-track etched polycarbonate membrane filters with ca. 80 nm in pore diameter, *D* and ca. 6 µm in pore length, *L* (ca. 75 in aspect ratio, *L*/*D*). Recently, we have reported that metallic nanowires can be electrodeposited in home-made AAO templates with ca. 33~85 nm in pore diameter, *D* and ca. 60 µm in pore length, *L* (ca. 700~1800 in aspect ratio, *L*/*D*) [23]. Hence, in this study, to reduce the effect of contact resistance between a lead wire and an electrodeposited multilayered nanowire, the electrochemical in-situ contact process was applied by using our home-made AAO templates ca. 75 nm in pore diameter, *D* and ca. 70 µm in pore length, *L* (ca. 933 in aspect ratio, *L*/*D*).

## 2. Materials and Methods

A cross-section of an aluminum rod 10 mm in diameter was electrochemically polished in an ethanol solution containing perchloric acid (20 vol.%) to achieve a mirror-like surface. During the electrochemical polishing process, cell voltage was kept at 50 V for 120 s. Afterwards, the polished cross-section of an aluminum rod was anodized in an aqueous solution containing oxalic acid (0.3 mol L^−1^) to form an AAO thick layer with numerous nanochannels. It is well known that the growth rate, pore diameter and pore ordering of AAO films depend on the anodization voltage (cell voltage) [24]. Lee et al. reported that there are two anodization voltage regions which enable to form the pore ordering in an oxalic acid solution [24]. One is 40 V (mild anodization process) and the other is 120~150 V (hard anodization process). On the other hand, a very small pore diameter will cause the diffusion limit of metal ions inside the nanochannels during the electrodeposition process, while a very large pore diameter will induce the formation of a magnetic layer with a multi-domain structure or a nanotube with a hollow structure. In this study, a pore diameter of ca. 70 nm was targeted to achieve an ideal multilayered structure without considering the formation of pore ordering. Hence, during this anodization process, cell voltage was kept at 70 V for 2 h.

After this anodization process, the AAO thick layer was exfoliated from the aluminum rod in an ethanol solution containing perchloric acid (50 vol.%). During this exfoliation process, cell voltage was kept at 75 V for 3 s [25]. These AAO films were used as a template for growing nanowires.

On the surface of the AAO films, a gold layer was sputter-deposited to cover the pores and to make a cathode, while a thin gold layer was also sputter-deposited not to cover the pore and to make an electrochemical in-situ contact with nanowires. A gold wire and an Ag/AgCl electrode were used as anode and reference electrode, respectively. An aqueous solution containing 0.5 M Co(SO_3_NH_2_)_2_·4H_2_O, 0.005 M CuSO_4_·5H_2_O and 0.4 M HBO_3_ was used as an electrolytic solution. Cathode polarization curves were measured over a wide range of potential to determine the optimum condition for the electrodeposition Cu and Co layers. Co/Cu multilayered nanowires with alternating Cu layer of ca. 1.2~2.0 nm and Co layer of ca. 11.8~36.9 nm in thickness were synthesized using a pulsed-potential deposition technique into an AAO nanochannels with large aspect ratio (75 nm in diameter and 70 µm in length). The growth rate of Co/Cu multilayered nanowires was estimated by a pore filling time, which was determined from a time-dependence of deposition current during the pulsed-potential deposition.

After the growing procedure, nanowires were released from AAO films by dissolving in an aqueous solution containing NaOH (5 mol L^−1^). The obtained nanowires were served as samples for transmission electron microscope (TEM, JEM-2010-HT) (JEOL Ltd., Tokyo, Japan) observation. Magnetic hysteresis loops and magnetoresistance hysteresis loops of the nanowires, which were embedded in AAO templates, were obtained using a vibrating sample magnetometer (VSM, TM-VSM1014-CRO) (Tamakawa Co., Sendai, Japan) and a DC voltage current source monitor (ADCMT 6242) (ADCMT, Saitama, Japan) with increasing the magnetic field up to 10 kOe. A magnetic field was applied to in-plane and perpendicular directions against to the membrane film plane. The perpendicular direction corresponds to the axial direction of nanowires.

## 3. Results and Discussions

### 3.1. Structure of Anodized Aluminum Oxide Membrane Filters

**Figure 1:**
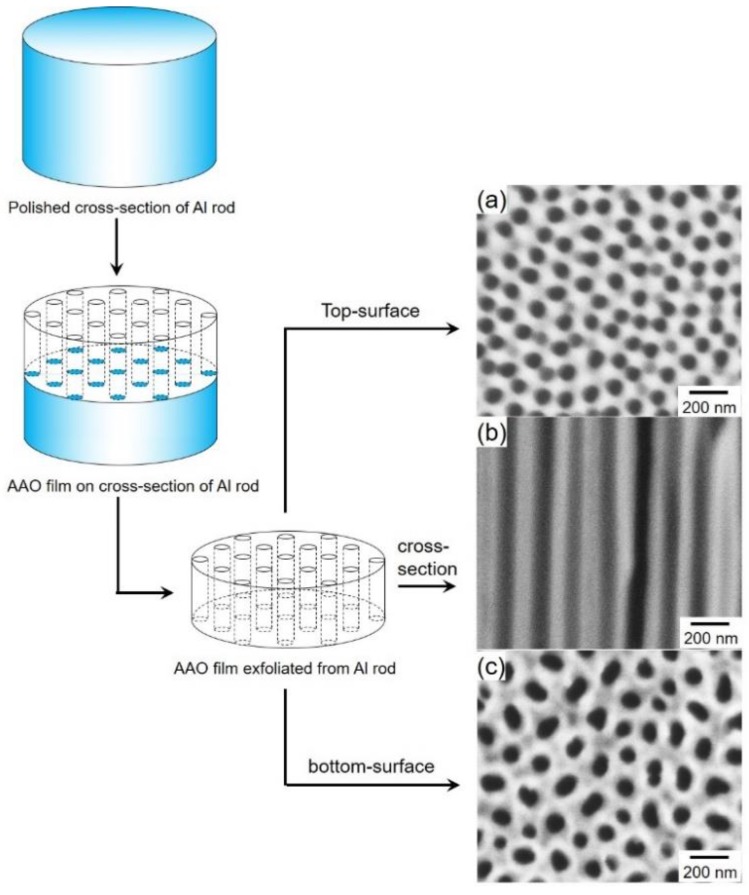
SEM images of top-surface (**a**), cross-section (**b**) and bottom-surface (**c**) of anodized aluminum oxide nanochannel templates that were anodized at 70 V for 2 h. The electrolytic solution temperature was kept at 25 °C during the anodization.

Figure 1 shows SEM images of top-surface (Figure 1a), cross-section (Figure 1b) and bottom-surface (Figure 1c) of an anodized aluminum oxide membrane filter exfoliated from a metallic aluminum rod. It was revealed that the exfoliated membrane filter had a typical porous columnar structure with an average channel diameter of ca. 75 nm and a channel density of ca. 10^8^ cm^−2^. The channel length, which corresponds to the oxide film thickness, was approximately 70 µm.

### 3.2. Electrodeposition Process of Co/Cu Multilayered Nanowires

**Figure 2:**
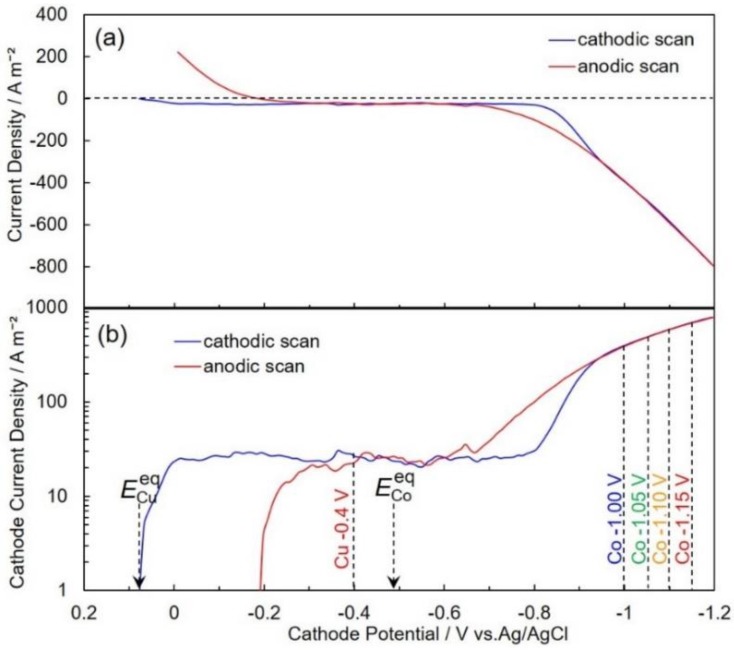
Cathodic and anodic scanned polarization curves for Cu and Co electrodeposition from an aqueous solution containing 0.5 M Co(SO_3_NH_2_)_2_·4H_2_O, 0.005 M CuSO_4_·5H_2_O and 0.4 M H_3_BO_3_. The solution temperature was kept at 40 °C. The observed current was plotted linearly (**a**) and logarithmically (**b**).

Figure 2 shows the cyclic voltammogram (Figure 2a) and Tafel plot of the cathodic polarization curve (Figure 2b) during the electrochemical reduction of Cu^2+^ and Co^2+^ ions. Based on an electrochemical theory, the equilibrium potential for Cu/Cu^2+^, *E*_Cu_^eq^, is calculated to be ca. +0.07 V vs. Ag/AgCl, while that for Co/Co^2+^, *E*_Co_^eq^, is also estimated to be around −0.48 V vs. Ag/AgCl. As shown in Figure 2a, the remarkable risings in the cathodic and anodic current density are observed in the potential of ca. −0.80 V and −0.30 V, respectively. Considering *E*_Co_^eq^, the cathodic and anodic current seem to be the reduction and dissolution current of Co^2+^ ions. To investigate the detail in electrochemical reduction behavior of Cu^2+^ ions, the Tafel plot was employed to enlarge the very small cathode current region. As shown in Figure 2b, the cathode current begins to increase at ca. −0.07 V, which corresponds to *E*_Cu_^eq^. Usually, in an acidic aqueous solution, Cu^2+^ ions are easily reduced to metallic state in the process without large overpotential. Therefore, this increase in the cathode current seems to correspond to an electrochemical reduction current of Cu^2+^ ions. When the cathode current density increases up to around 25 A m^−2^, the cathode potential polarizes to ca. −0.8 V. In this current density region, Cu^2+^ ions diffusion seems to reach the limit. Furthermore, in the potential region, which is less-nobler than *E*_Co_^eq^, the cathode current density increases again at around −0.8 V. Generally, in an acidic aqueous solution, Co^2+^ ions are reduced to metallic state in the process with large overpotential [26] due to the multi-step reduction process, which is proposed by Bockris et al. [27]. Accordingly, this increase in the cathode current seems to correspond to an electrochemical reduction current of Co^2+^ ions. Moreover, in the potential region, which is less-nobler than −1.2 V, the following phenomenon is observed. When the cathode current density increases more than 1000 A m^−2^, significant polarization of the cathode potential is observed because the diffusion of Co^2+^ ions reaches to the limit. Finally, based on the above electrochemical behavior, the cathode potential ranges for alternating electrodeposition of Cu layer and Co layer in the multilayered nanowires were decided to be −0.40 V and −1.00~−1.15 V, respectively.

**Figure 3:**
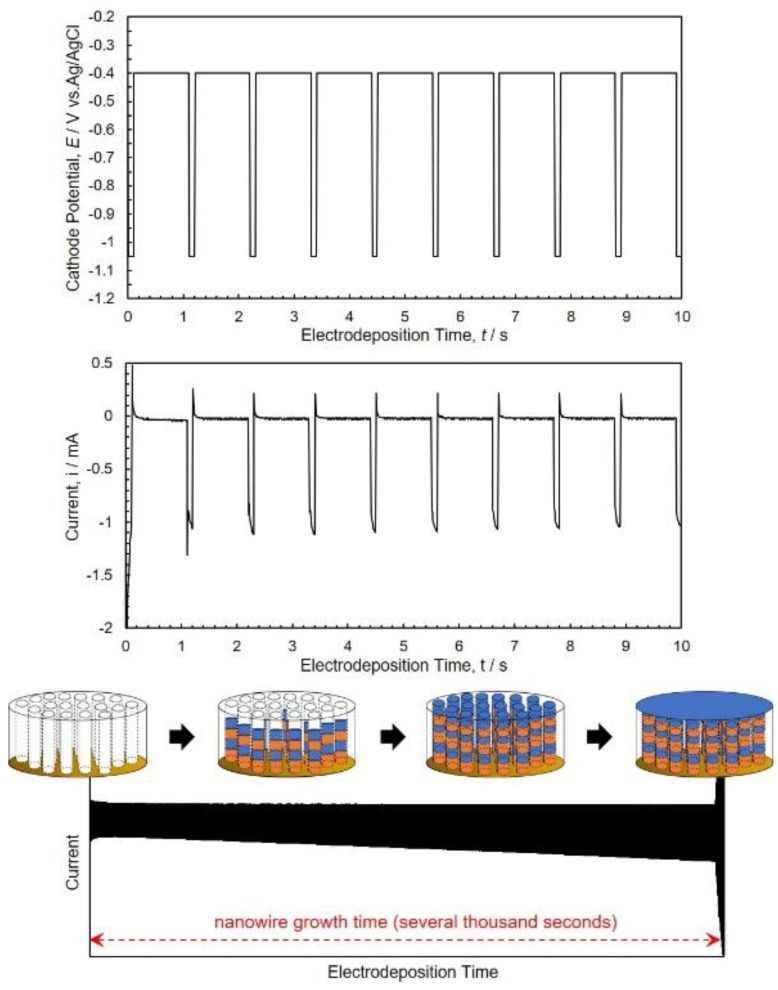
Time-dependence of observed current during the electrochemical in-situ contact process of Co/Cu multilayered nanowires in an anodized aluminum oxide nanochannel template. The cathode potential was alternatingly changed over between −0.4 V (for 1.0 s) and −1.05 V (for 0.1 s).

Co/Cu multilayered nanowires were synthesized by alternating the cathode potential from −0.40 V (for 1.0 s) to −1.00~−1.15 V (for 0.1 s) to control each layer’s thickness in a several nanometer scale. In order to determine the bilayer thickness of the Co/Cu layer in the multilayered nanowires, the filling time of AAO nanochannels with a length of 70 µm was determined by investigating the time-dependence of the observed current at each pulsed potential condition as shown in Figure 3. When the nanowires reach up to the membrane surface, the current will increase drastically due to the electrochemical in-site contact with Au layer on the AAO surface. Hence, the growth rates can be determined by dividing the channel length by the filling time. At the pulsed potential of −0.40 V for the Cu layer and −1.05 V for the Co layer, the growth rate was around 24 nm/s. In this growth condition, the bilayer thickness of the Co/Cu layer was estimated to be about 26 nm.

**Figure 4:**
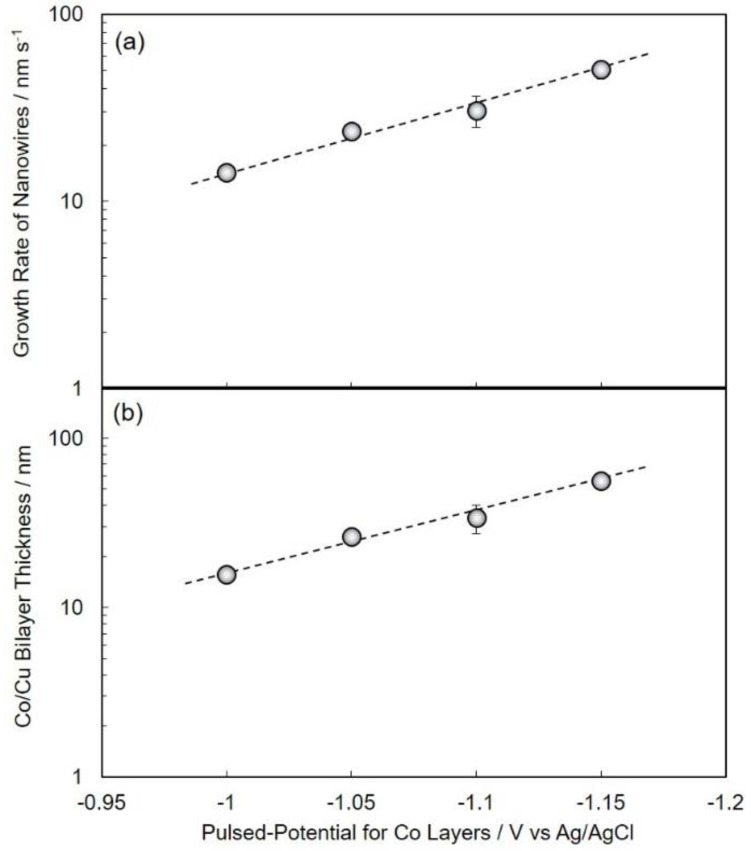
Effect of pulsed-potential for Co layers, *E*_Co_, on the growth rate of nanowires (**a**) and Co/Cu bilayer thickness (**b**).

**Figure 5:**
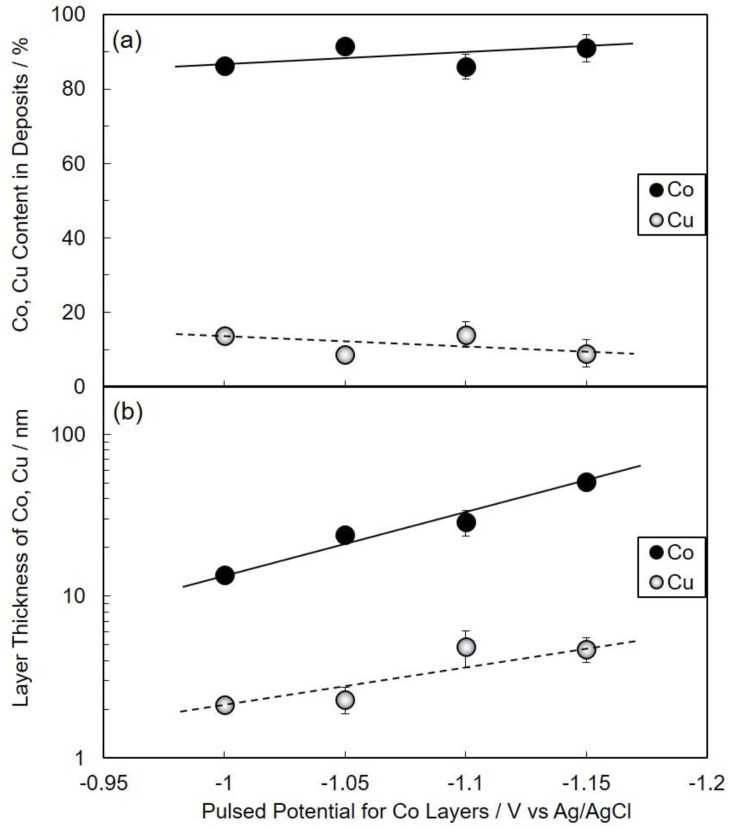
Effect of pulsed-potential for Co layers, *E*_Co_, on Co and Cu content in deposits (**a**) and layer thickness of Co and Cu (**b**).

Figure 4 shows the effect of pulsed-potential for the Co layer, *E*_Co_, on the nanowire growth rate (Figure 4a) and Co/Cu bilayer thickness (Figure 4b). With the shifting of *E*_Co_ to the less-noble region, as shown in Figure 4a,b, the nanowire growth rate and Co/Cu bilayer thickness increases up to ca. 51 nm/s and 56 nm, respectively. Figure 5 shows the effect of pulsed-potential for the Co layer, *E*_Co_, on the Co and Cu content in deposits (Figure 5a) and layer thickness of Co and Cu (Figure 5b). Over the potential range of *E*_Co_, the Co content was ca. 90%, while the Cu content was ca. 10% as shown in Figure 5a. On the other hand, with shifting *E*_Co_ to the less-noble region, the layer thickness of Co increases logarithmically up to ca. 51 nm, while the layer thickness of Cu seems to be an almost stable value ca. 2~4 nm as shown in Figure 5b.

### 3.3. Structure of Co/Cu Multilayered Nanowires

**Figure 6:**
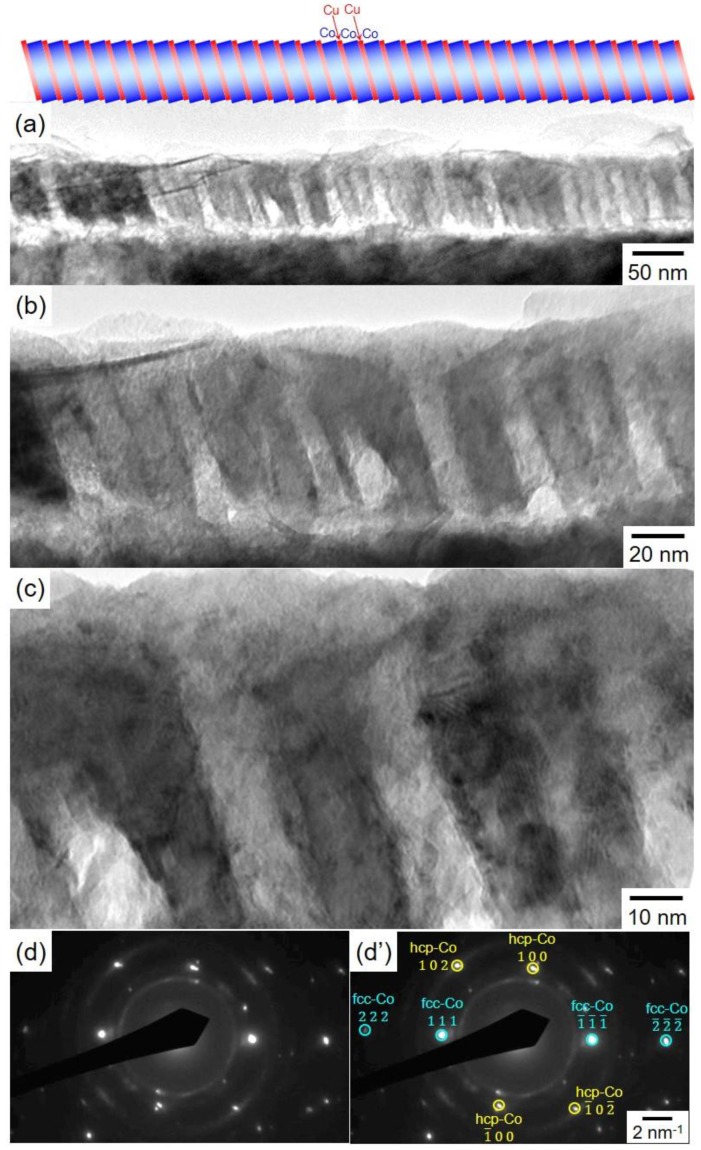
TEM images of a Co/Cu multilayered nanowire that was pulsed-potential deposited at −0.40 V for 1.0 s and −1.05 V for 0.1 s (**a**–**c**). The electron diffraction patterns are also shown in (**d**–**d’**). The Co/Cu multilayered nanowires were separated from an anodized aluminum oxide nanochannel template.

Figure 6 shows TEM images of a Co/Cu multilayered nanowire that was pulsed-potential deposited at −0.40 V for 1.0 s and −1.05 V for 0.1 s in a low magnification (Figure 6a), in a middle magnification (Figure 6b) and in a high magnification (Figure 6c). The electron diffraction patterns are also shown in Figure 6d. The Co/Cu multilayered nanowires were separated from an anodized aluminum oxide nanochannel template. As shown in Figure 6a, the diameter of a Co/Cu multilayered nanowire is ca. 75 nm which corresponds well to the nanochannel diameter of AAO template as shown in Figure 1a. The nanowire also has a multilayered structure with alternating thin layer of ca. 2 nm and thick layer of ca. 15 nm in thickness. As shown in Figure 5b, considering the estimation results in the layer thickness, the thin layer and the thick layer correspond to Cu and Co, respectively. Each interface between Cu and Co layer seems to be quite sharp and any intermixing or alloying layers are not observed. However, the normal direction of each layer seems to be slightly inclined to the axial direction of each nanowire. This inclination might be caused by the surface roughness of sputter-deposited Au layer. Furthermore, as shown in Figure 6d, bright spot pattens of hcp-Co (100), hcp-Co (102) and fcc-Co (111) are observed. Several other researchers also reported that the electrodeposited Co nanowires were composed of both the hcp and fcc crystallographic phases [28]. Based on a phase diagram of the Co-Cu binary alloy system, the solubility limit of Cu in fcc-Co alloy phase is larger than that in hcp-Co alloy phase. Hence, it is estimated that the phase transformation from hcp to fcc will be observed with an increase in Cu content of the alloy [29]. Therefore, in this study, electrodeposited fcc-Co phase seems to contain certain amount of Cu impurities.

**Figure 7:**
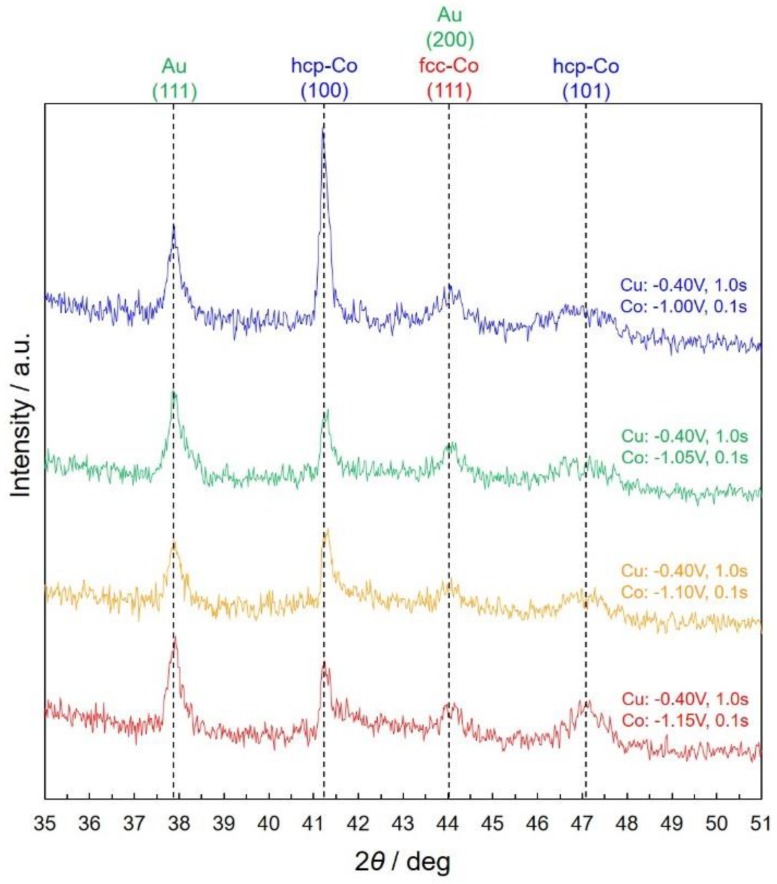
Effect of pulsed-potential for Co layer, *E*_Co_, on the X-ray diffraction patterns of electrodeposited Co/Cu multilayered nanowires. The pulsed-potential for Co layers were set for −1.00 V, −1.05 V, −1.10 V, −1.15 V.

Figure 7 shows the effect of pulsed-potential for Co layer, *E*_Co_, on the X-ray diffraction patterns of electrodeposited Co/Cu multilayered nanowires. According to Figure 7, the diffraction peaks at 41.25°, 44° and 47° are originated from hcp-Co (100), fcc-Co (111) and hcp-Co (101), respectively. These results correspond well to the electron diffraction spot-patterns in Figure 6d.

### 3.4. Magnetoresistance Properties of Co/Cu Multilayered Nanowires

**Figure 8:**
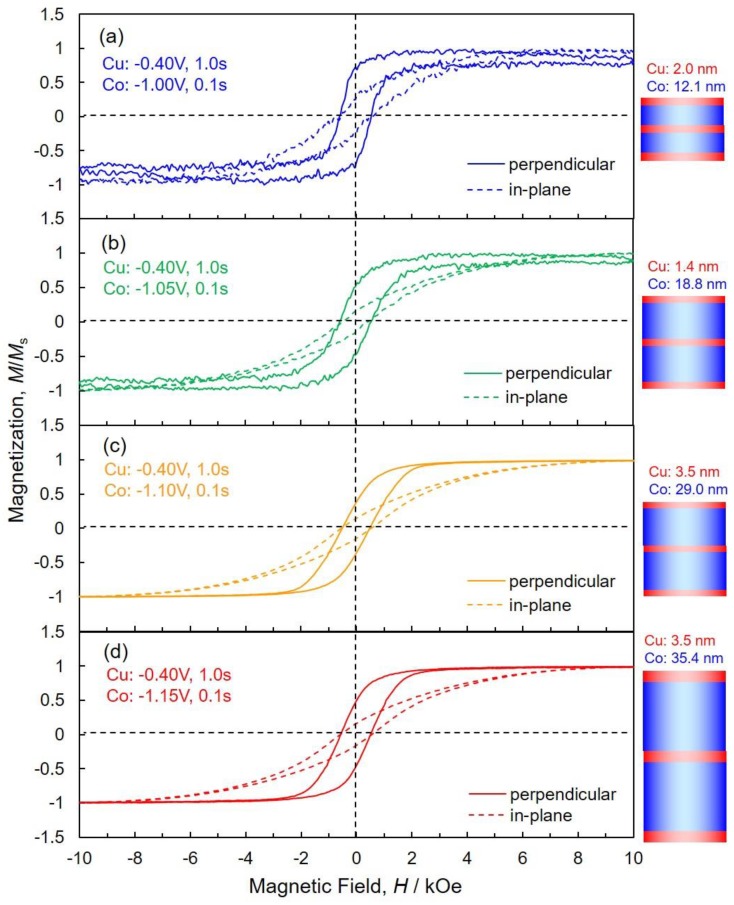
Effect of pulsed-potential for Co layers, *E*_Co_, on the magnetic hysteresis loops of anodized aluminum oxide (AAO) nanochannel films with Co/Cu multilayered nanowire arrays. *E*_Co_ was set at −1.00 V (**a**), −1.05 V (**b**), −1.10 V (**c**) and −1.15 V (**d**). A magnetic field was applied to perpendicular (solid lines) and in-plane (dotted lines) directions to the layers interface.

Figure 8 shows the effect of pulsed-potential for Co layers, *E*_Co_ on the magnetic hysteresis loops of AAO nanochannel films with Co/Cu multilayered nanowire arrays. Magnetic field was applied to perpendicular (solid lines) and in-plane (dotted lines) directions to the layers interface. As shown in dotted lines of Figure 8, Co/Cu multilayered nanowire arrays are hardly magnetized in the in-plane direction to the membrane film plane due to an extremely large demagnetizing field, *H_d_*, which is caused by the inverse magnetic poles generated on lateral faces of the nanowires. It is well-known that the effective magnetic field, *H_eff_* can be expressed by the following Equation (3).

(3) $$H_{eff}=H_{a}-H_{d}=H_{a}-f_{d}\frac{M}{\mu_{0}}$$

Here, *H_a_* is applied magnetic field, *f_d_* is materials shape factor in demagnetizing field, *M* is magnetic moment and *μ*_0_ is permeability constant. Furthermore, *f_d_* can be expressed by the following equations.

(4) $$f_{d}^{in}=\frac{1-f_{d}^{per}}{2}$$

(5) $$f_{d}^{per}=\frac{ln2k-1}{k^{2}}$$

Here, *f_d_^in^* is a materials shape factor in demagnetizing field when the magnetic field is applied to in-plane direction to the membrane film plane, while *f_d_^per^* is a factor when the magnetic field is applied to perpendicular direction to the plane. *k* is an aspect ratio of a nanowire. In the present work, *k* is 933. Hence, *f_d_^in^* is nearly 0.5 and *f_d_^per^* is almost zero, respectively. When the magnetic field is applied to in-plane direction to the plane, *f_d_^in^* is nearly 0.5 and is maximum, hence *H_eff_* will be minimum value. Therefore, in the present work, Co/Cu multilayered nanowires array was hardly magnetized in an in-plane direction to the membrane film plane due to the small *H_eff_*.

On the contrary, as shown in solid lines of Figure 8, Co/Cu multilayered nanowire arrays were easily magnetized in the perpendicular direction to the membrane film plane and the magnetization reaches to saturation at around 2 kOe. When the magnetic field is applied in the perpendicular direction to the membrane film plane, *f_d_^per^* is almost zero and minimum, hence *H_eff_* will be maximum value. Therefore, in the present work, Co/Cu multilayered nanowires array was easily magnetized perpendicular to the membrane film plane due to the large *H_eff_*, while the coercive force of Co/Cu multilayered nanowires array is less than 1 kOe. The effect of pulsed-potential for Co layers, *E*_Co_ on the magnetic properties are summarized in the following Table 1.

By the way, in a ferromagnetic nanowires array structure, magnetostatic dipole interaction field, *H_dp_* will be induced among the nanowires [30]. *H_dp_* can be expressed by the following Equation (6).

(6) $$H_{dp}=6.3M_{s}\frac{\pi r^{2}L}{D_{i}^{3}}$$

Here, *M_s_* is the saturation magnetization, *r* and *L* are the radius and length of the nanowires, respectively, *D_i_* is the interwire distance. Based on the Equation (6), *H_dp_* decreases with a decrease in *M_s_*. This results in enhancing the perpendicular magnetic anisotropy effect due to the shape anisotropy of individual nanowires. According to Figure 8a, the squareness, *M_r_/M_s_,* of Co/Cu multilayered nanowires array is ca. 0.69, that is higher than those of the others (Figure 8b–d). While the saturation magnetization, *M_s_,* of the sample (Figure 8a) is smaller than those of the others because the nonmagnetic Cu content is higher than those of the others as shown in Figure 5a. Hence, the perpendicular magnetic anisotropy effect of the sample (Figure 8a) seems to be enhanced by the smaller magnetostatic dipole interaction field than those of the others.

Furthermore, in a multilayer structure with ferromagnetic layers and nonmagnetic layers, it has been reported that antiferromagnetic coupling behavior can be observed in the magnetization process [31]. However, in this study, such characteristic coupling behavior was not observed in the electrodeposited Co/Cu multilayered nanowires array.

**Figure 9:**
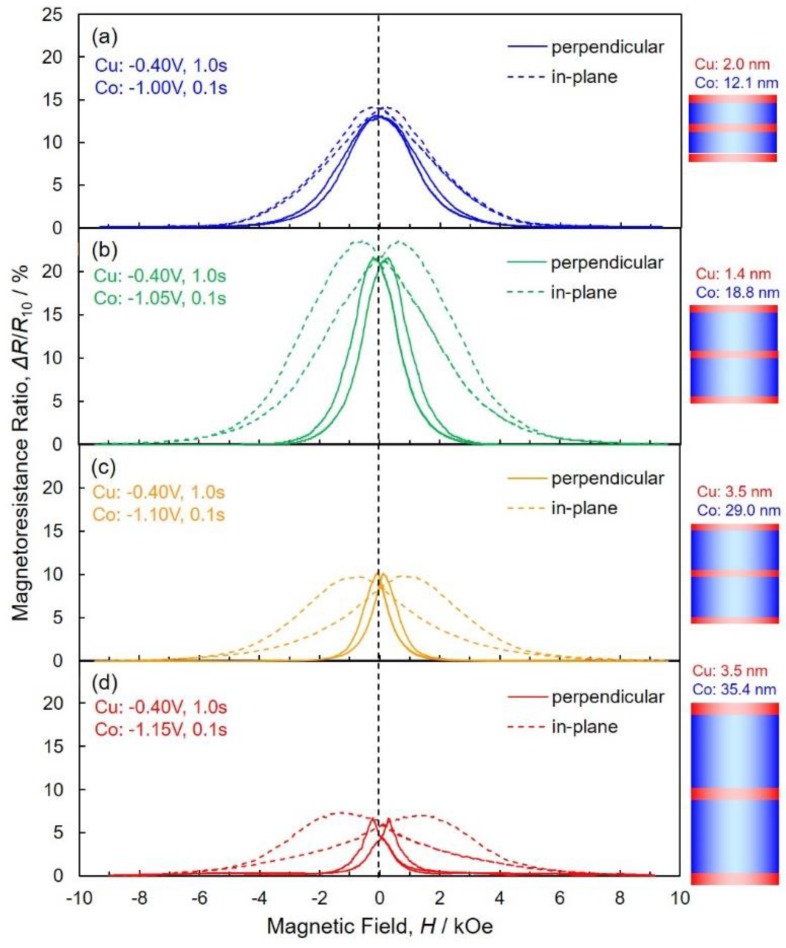
Effect of pulsed-potential for Co layers, *E*_Co_, on the magnetoresistance hysteresis loops of AAO nanochannel films with Co/Cu multilayered nanowire arrays. *E*_Co_ was set at −1.00 V (**a**), −1.05 V (**b**), −1.10 V (**c**) and −1.15 V (**d**). A magnetic field was applied to perpendicular (solid lines) and in-plane (dotted lines) directions to the layers interface.

Figure 9 shows the effect of pulsed-potential for Co layers, *E*_Co_, on the magnetoresistance hysteresis loops of AAO nanochannel films with Co/Cu multilayered nanowire arrays. A magnetic field was applied to perpendicular (solid lines) and in-plane (dotted lines) directions to the layers interface. As shown in the dotted lines of Figure 9, the magnetoresistance ratio of Co/Cu multilayered nanowire arrays are gradually decreased with an increase in the magnetic field and reaches zero at ca. 8 kOe. On the contrary, as shown in the solid lines of Figure 9, the magnetoresistance ratio of Co/Cu multilayered nanowire arrays are rapidly decreased with an increase in the magnetic field and reaches zero at ca. 2 kOe.

**Figure 10:**
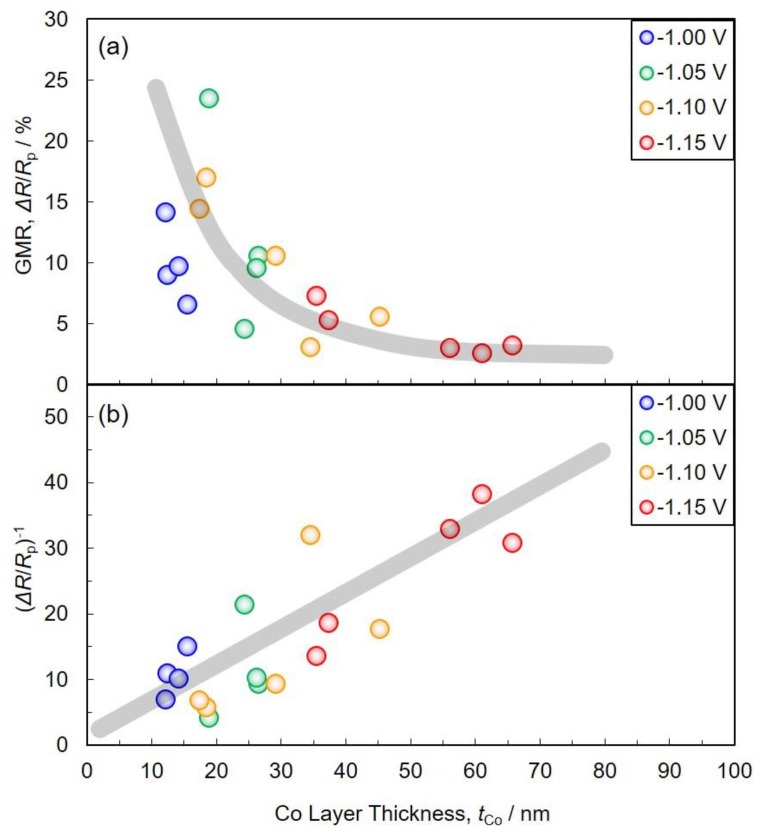
Effect of Co layer thickness on GMR (**a**) and (Δ*R*/*R_p_*)^−1^ (**b**) in electrodeposited Co/Cu multilayered nanowires.

Figure 10 shows the effect of Co layer thickness on GMR (a) and (Δ*R*/*R_p_*)^−1^ (b) in electrodeposited Co/Cu multilayered nanowires. As shown in Figure 10a, with a decrease in Co layer thickness, the GMR value increases up to ca. 23.5% in the layer thickness of ca. 18.8 nm.

According to the Valet–Fert model [32], in the conditions of *t_N_* < *l_N_^sf^* and *t_F_* > *l_F_^sf^*, (Δ*R*/*R_p_*)^−1^ varies linearly with *t_F_* as given by the following Equation (7). Here, *t_N_* and *t_F_* are the thickness of nonmagnetic and ferromagnetic layers, respectively. *l_N_^sf^* and *l_F_^sf^* are the spin diffusion length of nonmagnetic and ferromagnetic layers, respectively. Δ*R* is the difference between *R_ap_* and *R_p_*. *R_ap_* is the resistance of a multilayer with the magnetization vectors of successive ferromagnetic layers aligned antiparallel while *R_p_* is the corresponding resistance with them aligned parallel.

(7) $${\left(\frac{\Delta R}{R_{p}}\right)}^{-1}=ct_{F}$$

In the above Equation (7), *c* is defined as the following equation.

(8) $$c=\frac{1-\beta^{2}}{2p\beta^{2}l_{F}^{sf}}$$

Here, *β* is the bulk spin asymmetry coefficient. *p* is the fraction of consecutive magnetic layers that have magnetizations aligned antiparallel.

As shown in Figure 10b, with a decrease in Co layer thickness, the (Δ*R*/*R_p_*)^−1^ value decreases linearly. This result can be explained by the Valet–Fert model as shown in Equation (7). Hence, *l_N_^sf^* will be longer than ca. 5 nm, while *l_F_^sf^* will be shorter than ca. 12 nm.

## 4. Conclusions

AAO thick films (ca. 70 µm in length) with numerous nanochannels (ca. 10^8^ cm^−2^ in channel density) were synthesized by an anodization and an exfoliation process on a cross-section of a metallic aluminum rod. The average channel diameter was ca. 75 nm and the aspect ratio reached ca. 933. Co/Cu multilayered nanowires were synthesized by using a pulsed-potential deposition technique from an aqueous solution. During the electrodeposition of Co/Cu multilayered nanowires, the cathode potential was alternated from −0.40 V (for 1.0 s) to −1.00~−1.15 V (for 0.1 s). The growth rate of the multilayered nanowires ranged from ca. 14 to 51 nm/s. Co/Cu multilayered nanowires were composed of both hcp-Co and fcc-Co phase. Co/Cu multilayered nanowire arrays were easily magnetized in the perpendicular direction to the membrane film plane due to their extremely large aspect ratio. With decreasing Co layer thickness, the GMR value increased up to ca. 23.5% and this tendency was able to be explained by the Valet–Fert model in the condition of *t_N_* < *l_N_^sf^* and *t_F_* > *l_F_^sf^*.

## Figures and Tables

**Table 1:** Magnetic properties of electrodeposited Co/Cu multilayered nanowires array.

*E*_Co_/V	*t*_Co_*/*nm	*H_c_* (in-Plane)/kOe	*H_c_* (Perpen.)/kOe	*M_r_*/*M_s_* (in-Plane)	*M_r_*/*M_s_* (Perpen.)
−1.00	11.8	0.56	0.62	0.27	0.69
−1.05	19.3	0.48	0.59	0.17	0.55
−1.10	20.2	0.57	0.53	0.15	0.39
−1.15	36.9	0.57	0.58	0.16	0.50
